# A narrative review of circulating tumor cells clusters: A key morphology of cancer cells in circulation promote hematogenous metastasis

**DOI:** 10.3389/fonc.2022.944487

**Published:** 2022-08-18

**Authors:** Qiong Chen, Jueyao Zou, Yong He, Yanhong Pan, Gejun Yang, Han Zhao, Ying Huang, Yang Zhao, Aiyun Wang, Wenxing Chen, Yin Lu

**Affiliations:** ^1^ Jiangsu Key Laboratory for Pharmacology and Safety Evaluation of Chinese Materia Medica, School of Pharmacy, Nanjing University of Chinese Medicine, Nanjing, China; ^2^ Department of Pharmacy, The Second Affiliated Hospital of Nanjing Medical University, Nanjing, China; ^3^ Jiangsu Collaborative Innovation Center of Traditional Chinese Medicine (TCM) Prevention and Treatment of Tumor, Nanjing, China

**Keywords:** circulating tumor cells, CTC cluster, homotypic type, heterotypic type, hematogenous metastasis

## Abstract

Circulating tumor cells (CTCs) that survive in the blood are playing an important role in the metastasis process of tumor. In addition, they have become a tool for tumor diagnosis, prognosis and recurrence monitoring. CTCs can exist in the blood as individual cells or as clumps of aggregated cells. In recent years, more and more studies have shown that clustered CTCs have stronger metastasis ability compared to single CTCs. With the deepening of studies, scholars have found that cancer cells can combine not only with each other, but also with non-tumor cells present in the blood, such as neutrophils, platelets, etc. At the same time, it was confirmed that non-tumor cells bound to CTCs maintain the survival and proliferation of cancer cells through a variety of ways, thus promoting the occurrence and development of tumor. In this review, we collected information on tumorigenesis induced by CTC clusters to make a summary and a discussion about them. Although CTC clusters have recently been considered as a key role in the transition process, many characteristics of them remain to be deeply explored. A detailed understanding of their vulnerability can prospectively pave the way for new inhibitors for metastasis.

## Introduction

As we all know, the leading cause of death in patients with cancer diseases is the formation of metastatic tumors. Deaths due to metastatic tumors account for ninety percent of cancer-related deaths (1). Tumor metastasis is a multi-step process of cell biology. It can be succinctly summarized as the progress that cancer cells shed from the primary solid tumor reach the distal metastasis through a series of processes, then adapt to foreign tissue microenvironment, and finally form secondary tumor sites (2). In the whole process, cancer cells can break off from the solid tumor lesion at the primary or metastatic site. Then they enter the bloodstream, which is the main cause of metastatic tumors (3). Circulating tumor cells (CTCs) refer to all kinds of cancer cells in peripheral blood (PB). As early as 1869, Thomas discovered the existence of CTCs in patients with tumor diseases. Today, the analysis of blood for CTCs called “liquid biopsy” has opened new avenues for cancer surveillance (4). If there is no CTCs detected in the blood sample, it can be judged that the patient has a good prognosis (5). In fact, CTCs can not only exist as single cells, but also aggregate into multicellular population in PB. A cell cluster formed by the aggregation of two or more cancer cells through intercellular adhesion molecules is called a CTC cluster (6). Cancer cells can fall off from the primary site in clumps, and at the same time, it has been documented that individual cancer cells in the blood system can also clump together to form CTC clusters (7). As early as 1954, Watanabe found that CTC clusters were of paramount importance for metastatic tumors (8). In the following decades, continuous studies have revealed that metastatic tumors are significantly correlated with the size and number of CTC clusters (9, 10). However, separating and enriching CTCs and CTC clusters from fresh blood samples have been a tough problem to overcome for a long time, so further researches on them have stalled for decades.

With the unceasing development of science and technology, considerable number of specialized CTC isolation instruments have been invented in order to better detect CTCs, such as CellSearch®, CTC–Chip, CTC–iChip, etc (11). The principle of separating CTCs from blood can be divided into two parts: one is based on the physical properties of CTC clusters and the other is based on immunofluorescence biomarker staining. These methods have greatly promoted the pace of researches on the molecular and biological characteristics of CTC clusters. In recent years, it has been wildly reported that the ability of CTC clusters to metastasize and proliferate is stronger than that of individual CTCs (12). Moreover, there are complex interactions between non–tumor cells in blood circulation and cancer cells, and non–tumor cells combined with CTCs include neutrophils, myeloid–derived suppressor cells (MDSCs), tumor–associated macrophages (TAMs), platelets and cancer–associated fibroblasts (CAFs), etc. These non–tumor cell scan promote tumor metastasis in many ways (12). Now more and more people are concentrating on studying CTC clusters intently and CTC clusters may even represent a valuable and promising new treatment option.

## Strong metastatic potential of CTC clusters

More than 100 years ago, Stephen Paget proposed the hypothesis of “seeds and soil”. In this hypothesis, CTCs are considered as the “seeds” of metastatic tumors, whose growth and colonization depend on the environment of the surrounding soil (13). It should be noted that CTC clusters exist in and are closely associated with many types of cancer, including liver cancer, lung cancer, colorectal cancer, renal cancer and pancreatic cancer, especially breast cancer (14–16). A recent study on prostate cancer demonstrated that the metastasis from the primary lesion to the secondary site was a process of polyclonal diffusion. This suggests that the metastasis of CTCs is characterized by polyclonal transfer, and different polyclonal genes have disparate traits for growth and treatment response (17). In other words, CTCs circulate in the blood in the form of aggregated cell clusters, namely CTC clusters. Actually, CTCs and CTC clusters are extraordinarily rare in PB. There is only one CTC in a billion blood cells, and CTC clusters are even rarer, accounting for about three percent of whole CTCs (5). However, according to relevant tests, the probability of single CTCs successfully forming a metastasis is extremely small. Meanwhile, CTC clusters are considered as “seeds with soil”, so scholars believe that “seeds” carrying “soil” may be more able to metastasize and grow.

**Figure 1:**
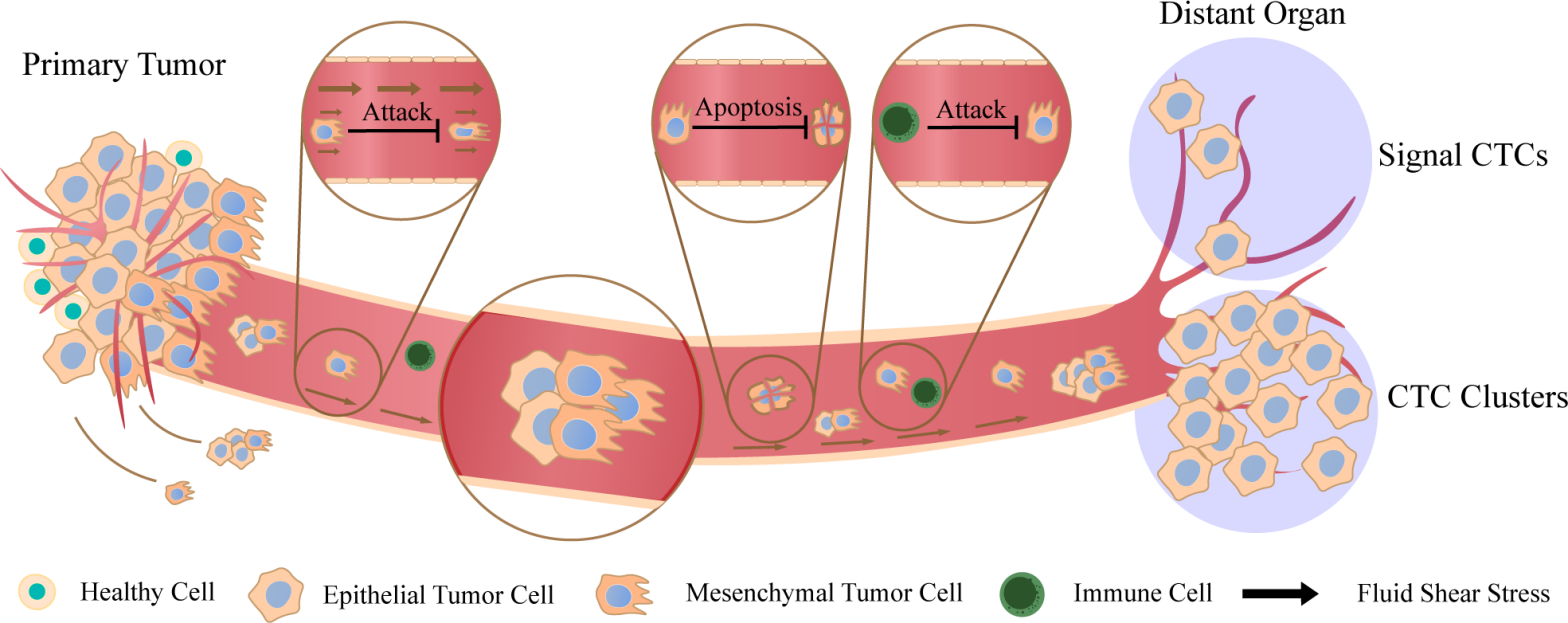
Metastasis of circulating tumor cells (CTCs) and CTC clusters Cancer cells fall off from the primary sites and enter the blood circulation. They can exist by the form of single cells or aggregated cell clusters. There are various factors in peripheral blood (PB) that are not conducive to the survival of cancer cells. Cancer cells will be attacked by fluid shear stress and immune system, and signal CTCs also induce apoptosis due to the loss of intercellular contact. Finally, they reach distant organs and continue to spread, and CTC clusters have stronger metastatic potential compared to signal CTCs.

Metastasis is a greatly complex process. First, cancer cells grow continuously and form tumor–related vessels. Then, cancer cells invade blood vessels through epithelial–mesenchymal transition (EMT) and enter the blood circulation. During EMT, epithelial bio–markers such as keratin, E–cadherin and epithelial cell adhesion molecule (EpCAM) are down–regulated while vimentin, N–cadherin, Snail1 (Snail), Snail2 (Slug) and other markers of mesenchymal cells are up–regulated (18). Quantitative 3D assessment of tumor budding at the cancer–host interface suggested that the migration of collective cells was considered as the mechanism of invasion, while the migration of single cancer cells seemed to be virtually absent (19). Then cancer cells in blood vessels eventually invade distant organs, where they colonize and proliferate to form tumor metastasis sites (20). As cancer cells travel through the blood, they will undergo various external pressures until they reach the distant organs, such as fluid shear stress (FSS), the attack of immune system and anoikis (21). Anoikis is defined as a unique phenomenon. The cancer cells in the primary sites adhere closely to each other or to the extracellular matrix. When they are separated from the primary sites individually, the cancer cells will apoptosis (22). It is a key mechanism that prevents cancer cells from growing and attaching to the matrix, thus avoiding the colonization of cancer cells in distant organs (23). More interestingly, in the original sites, cancer cells will form an immunosuppressive microenvironment to resist the damage of the immune system to them. So when they enter the blood system, they will lose the protection of immunosuppressive microenvironment. Therefore, only those cancer cells that can survive in the blood stream, adapt to the new environment of distant tissues, and induce angiogenesis can successfully spread and metastasize ultimately (24). Studies in vivo showed that CTC clusters were 50 times more likely to facilitate the metastasis than single CTCs (5). As the disease worsens, the number of detectable CTC clusters is increasing which shows that CTC clusters are closely related to the occurrence and development of cancer (25). This means that CTC clusters have more advantages in the process of tumor formation than individual cancer cells as **Figure 1** shows. At the same time, the question that why aggregated CTCs have stronger metastatic ability is being gradually explored.

## Homotypic circulating tumor cell clusters

**Figure 2:**
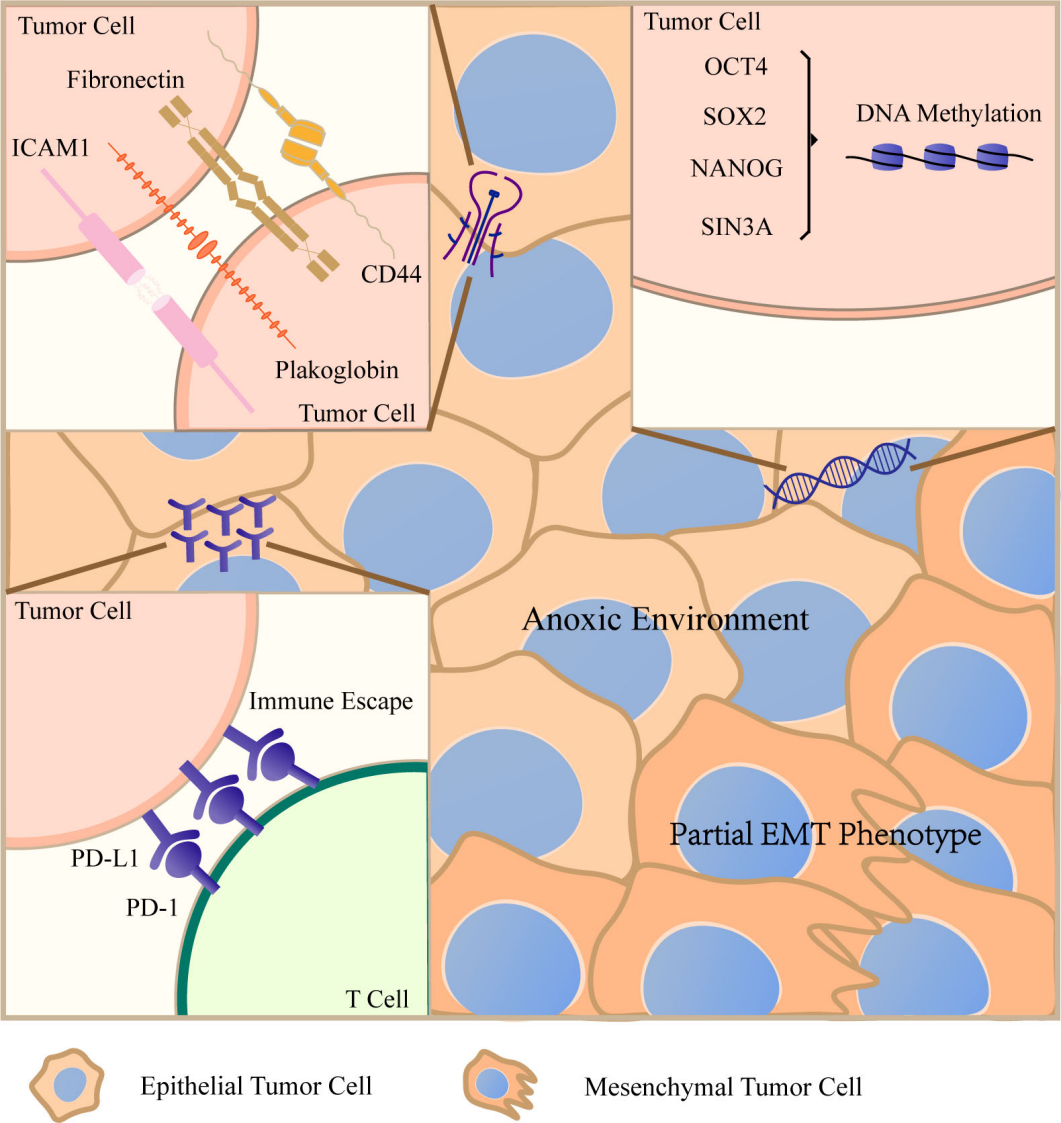
Characteristics of homotypic circulating tumor cell (CTC) clusters After CTCs gather into CTCT clusters, they will create favorable living conditions conducive to their own survival in a variety of ways. Cancer cells maintain important intercellular junction molecules, such as CD44, ICAM1, plakoglobin and so on, to resist anoikis. The aggregation of cancer cells can lead to hypomethylation of transcription factor binding sites related to stem cells and proliferation. CTC clusters keep hypoxic environment and the expression of PD–L1 increases significantly. They evade immune surveillance by these two ways. In addition, CTC clusters have a mixed epithelial/mesenchymal phenotype. When encountering small diameter vessels, they will be arranged in chains. All these characteristics lead to the stronger metastatic ability of CTC clusters.

CTC clusters can be roughly divided into two types according to the categories of composed cells. One is a kind of homotypic CTC clusters, and the other is heterotypic CTC clusters (26). When cell clusters contain two or more cancer cells, they are defined as homotypic CTC clusters (27). It has been found that when single or aggregated breast cancer cells were introduced into the tail vein of mice, aggregated cancer cells were more resistant to anoikis than single cells and their ability to implant in lung is also enhanced (28). Beyond that, many biological characteristics and advantages of promoting tumor metastasis have been revealed before. In the following part, we will display the main features of homotypic CTC clusters in **Figure 2**.

### CD44 mediates the adhesion and migration of CTCs

CD44 is a transmembrane glycoprotein and participates in cell proliferation, cell differentiation, cell migration, angiogenesis, docking of proteases on cell membranes (29). CD44 expression is up–regulated in subpopulations of cancer cells and also recognized as a molecular marker for CSCs (30). In 2015, Transmembrane 4 six family members 5 (TM4SF5) was found to bind and interact with CD44, thus improving the self–renewal properties of cancer cells and subsequently promoting the metastatic potential of CTCs (31). In 2019, Huiping Liu uncovered that aggregated cancer cells highly expressed CD44 and the results showed enhanced tumorigenesis and polyclonal metastasis of CTC clusters. Moreover, a mechanism for the formation of CTC clusters through CD44–PAK2 was demonstrated, which could further activate focal adhesion kinase (FAK), and FAK signal has been reported to control the adhesion, migration and proliferation of cancer cells (7). Epidermal growth factor receptor (EGFR) is a type I receptor tyrosine kinase. Together with its ligand, EGFR is involved in the regulation of a variety of cellular pathways. Knock–downing EGFR has been reported to inhibit cell growth (32). More interestingly, EGFR can enhance the CD44–mediated cancer cells aggregation and CD44 can stabilize EGFR in turn. Blocking EGFR by anti–EGFR monoclonal antibody could effectively prevent the aggregation of cancer cells and reduce the pulmonary metastasis of tumor. This implies that there is a positive feedback between EGFR and CD44 to maintain the stability of them (33).

### ICAM1 mediates the adhesion and transendothelial migration of CTCs

In 2021, Huiping Liu found that the expression of inter–cellular adhesion molecule 1 (ICAM1) increased 20 times in pulmonary metastatic cancer cells compared with non–metastatic cancer cells (28). ICAM1 is a cell surface glycoprotein typically expressed on endothelial cells and certain leukocytes that participates in many important processes, including cell signaling, cell–cell interaction, cell polarity and so on (34). CTCs can express ICAM1 on the surface of cell membrane and ICAM1 positive cells have stronger tumorigenic ability (35). The research showed that ICAM1 was not only a key promoter of the aggregation of homotypic CTC clusters, but also drove heterotypic adhesions between cancer cells and endothelial cells. What is more important, ICAM1 can maintain the level of cyclin–dependent kinase 6 (CDK6) and other signaling pathway components associated with the cell cycle, stemness, and survival (28). The extra–cellular matrix degrading enzyme heparinase (HPSE) is a gene associated with immune infiltration and cancer prognosis, and it can mediate the homotypic aggregation of CTCs in patients with breast tumor (36). Overexpression of HPSE can contribute to the aggregation of CTCs, and induce FAK signaling pathway and ICAM1–dependent cell adhesion, finally promoting the aggregation of cancer cells in the blood vessels (37).

### Plakoglobin is beneficial to the aggregation of CTCs

Plakoglobin is an important component of adhesion junctions and desmosomes. It can directly bind to E–cadherin and play an important role between desmosomal cadherin and interfilament cytoskeleton (38). In the beginning, plakoglobin was thought to be a tumor suppressor gene (39). Subsequently, plakoglobin was shown to be associated with the up–regulation of the PI3K/AKT and MAPK signaling pathways in the tumor microenvironment, which was beneficial to the occurrence and development of tumors (40). Single–cell RNA sequencing showed that the expression of plakoglobin in CTC clusters in breast tumor patients was 219 times higher than that in single CTCs. When plakoglobin was silenced in breast cancer cell lines, the likelihood of pulmonary metastasis was reduced in animal models. Results from the orthotopic xenograft model showed that silencing plakoglobin significantly reduced the number of aggregated CTCs, although neither the growth rate nor the number of single CTCs in the primary tumor was affected (5). This suggests that plakoglobin targets CTC clusters, promoting tumor metastasis by maintaining the aggregation of CTCs. According to the clinical analysis report, plakoglobin expression is an independent prognostic factor in breast cancer patients in clinical practice. Especially for overall survival, patients with high plakoglobin expression have lower survival rate (38).

### Other important mediators of intercellular adhesion

In addition to plakoglobin, CD44 and ICAM1 mentioned above, which play important roles in homotypic cell adhesion recognized by public, many other substances also participate in the aggregation of cancer cells. According to the relevant literature fibronectin plays an important role in the aggregation of CTCs (41). Further researches found that compared with normal cells, cancer cells could induce the upregulation of fibronectin upon detachment, which promoted the formation of CTC clusters and improved the ability to resist anoikis (42). Besides, CEACAM6 expressed on cancer cells can also help enhance the resistance of cancer cells to anoikis. In other words, CEACAM6 can maintain the adhesion between cancer cells (43). CTC clusters often express epithelial cytoskeleton protein, keratin 14 (K14). The result of RNA–seq analysis showed that the highly invasive K14 positive cells were rich in desmosome and hemi–desmosome adhesion complex genes. This indicates that there is a strong correlation between K14 and cell adhesion (44). Recently, two studies have shown that claudin 3 and claudin 4 are both extremely important proteins in the formation of CTC clusters (45, 46). In general, there are many substances and regulatory mechanisms to maintain the aggregation of cancer cells.

### Mechanical deformation

Throughout the tumor metastasis process, CTC clusters have obviously more advantages in the stage of hematogenous metastasis than single CTCs. Although the volume of CTC clusters is very large and they may not penetrate micro–vessels easily, relevant literature reported that more than ninety percent of CTC clusters with as many as 20 cells could successfully penetrate 5 μm to 10 μm blood vessels in whole blood. CTC clusters move quickly and reversibly form single–stranded cells, which greatly reduces the hemodynamic resistance that they are subjected to. After extrusion, the nucleus of CTC clusters may deform from circular to slender ellipsoid. Morphological changes involve the whole cell clusters and the cell itself (47). In addition, cancer cells can soften themselves during the migration, which is the adaptation mechanism of their mechanical means of invasion (48). At the same time, their large volume is their physical advantage as well, mainly because when they are easily trapped in the blood vessels of distal metastatic organs, cell–cell adhesion can enhance the ability of cancer cells to metastasize. To sum up, mechanical morphological changes and tight junctions between cancer cells make CTC clusters with strong metastatic ability (26).

### DNA hypomethylation

There is a significant correlation between cancer progression and DNA methylation. It has previously been reported that tumor–associated methylation can be detected in CTCs (49). A comprehensive analysis of CTC methylome identified a unique DNA methylation signature of CTCs. Further analysis showed that methylation of promoters of epithelial genes was related to the EMT process (50). In 2011, the first report on epigenetic changes in CTCs was published. It indicated that DNA hypermethylation of tumor suppressor gene was a marker of CTCs in breast cancer patients (51). In 2019, CTC clusters have been reported to be related to DNA methylation kinetics. Clusters of cancer cells can lead to hypomethylation of binding sites for transcription factors associated with stem cells and proliferation. When CTC clusters were dissociated into single cells, the associated transcription factor binding sites would remethylate, including OCT4, SOX2, NANOG and SIN3A. As a result, cancer cells were less able to spread and metastasize (52). By contraries, the activation of OCT4, EGFR, and PAK2/FAK signaling pathway can mediate homotypic aggregation of CTCs and further promote homologous interaction of CD44 (7).

### Immune escape

The activation of T cells plays an important role in the immune regulation of the body and T cells are highly lethal to cancer cells. Programmed cell death 1 (PD–1) is a negative immunomodulatory checkpoint expressed on activated T cells. It plays a core role in adaptive cellular immunity and controls the activation and differentiation of T cells (53). Programmed cell death ligand 1 (PD–L1) is expressed by cancer cells. When PD–L1binds to PD–1, it can strongly inhibit signal transmission to T cells, thus affecting cytokine production and inhibiting the proliferation of T cells (54). Studies have shown that increased expression of PD–L1 on cancer cells has the ability of leading to reduced tumor–specific immunity (55). A recent research showed that PD–L1 was often expressed in CTCs, so CTCs can evade immune surveillance in this way. The detection of CTCs expressing PD–L1 indicates the weakening of immune defense mechanism and the development of metastatic tumors, ultimately leads to poor prognosis of patients (56). Moreover, compared with single CTCs, the expression of PD–L1 was higher in CTC clusters. This means that CTC clusters are more tolerant to the damage of the immune system. Therefore, the possibility of forming metastatic tumors of CTC clusters will become higher (57).

### Partial EMT phenotype

EMT progression plays an important role in metastatic tumors. Morphological and molecular characteristics are used to characterize EMT states, including loss of cell–cell adhesion, changes of cell shape from round to oval shape, increased migration/invasion ability and improved resistance to anoikis (58). Studies have shown that the partial epithelial/mesenchymal phenotype of CTC clusters is an important means for them to make a living in human bodies (59). Weakly migrated subsets are more epithelial subsets and highly migrated subsets are more mesenchymal subsets (60). CTC clusters exhibit a partial EMT phenotype, with some cells in the cluster acting as “leaders” (on the first line of migration) and others as “followers”. EMT phenotype is more evident in “leader” cells (61), and TGF–β–SMAD signaling pathway has been reported to play an important role in metastasis. Blocking TGF–β can inhibit the expression of E–cadherin, causing cancer cells to partially or completely lose the E–cadherin–mediated adhesion connection, while enhancing their motility and invasiveness (21). The “alternating” mesenchymal phenotype and epithelial phenotype are particularly helpful in tumor progression and enhances their plasticity.

### Internal anoxic environment

Hypoxia has a very close relationship with angiogenesis, immune evasion, CSCs characteristics, etc (62). It is an important feature of tumor survival environment, which can induce EMT to contribute to the movement of cancer cells (63). Hypoxia–inducible factor 1 alpha (HIF–1α) is overexpressed in human cancers, partly as a result of hypoxia within tumors (64). On the other hand, compared with primary tumors, HIF–1α protein expression in metastatic tumors is increased, while hypoxia signaling in CTCs predicts reduced overall survival in patients with cancer metastasis (65). When cancer cells gather together, they can induce an anoxic environment to form an immunosuppressive microenvironment suitable for their growth (66). On the other hand, internal anoxic environment is the main reason for the enhancement of cell–cell adhesion and the formation of CTC clusters, and the hypoxic CTC clusters are more likely to invade blood vessels (67). High expression of Desmoglein2 (DSG2) has the huge potential for promoting tumor growth, increasing the prevalence of CTC clusters, and facilitating distant organ colonization. More importantly, the regulation mediated by DSG2 of CTC clusters is affected by upstream HIF–1α (68). So this means that CTC clusters maintain the hypoxic environment, and in turn, the hypoxic environment maintains the survival of cancer cells.

## Heterotypic circulating tumor cell clusters

**Figure 3:**
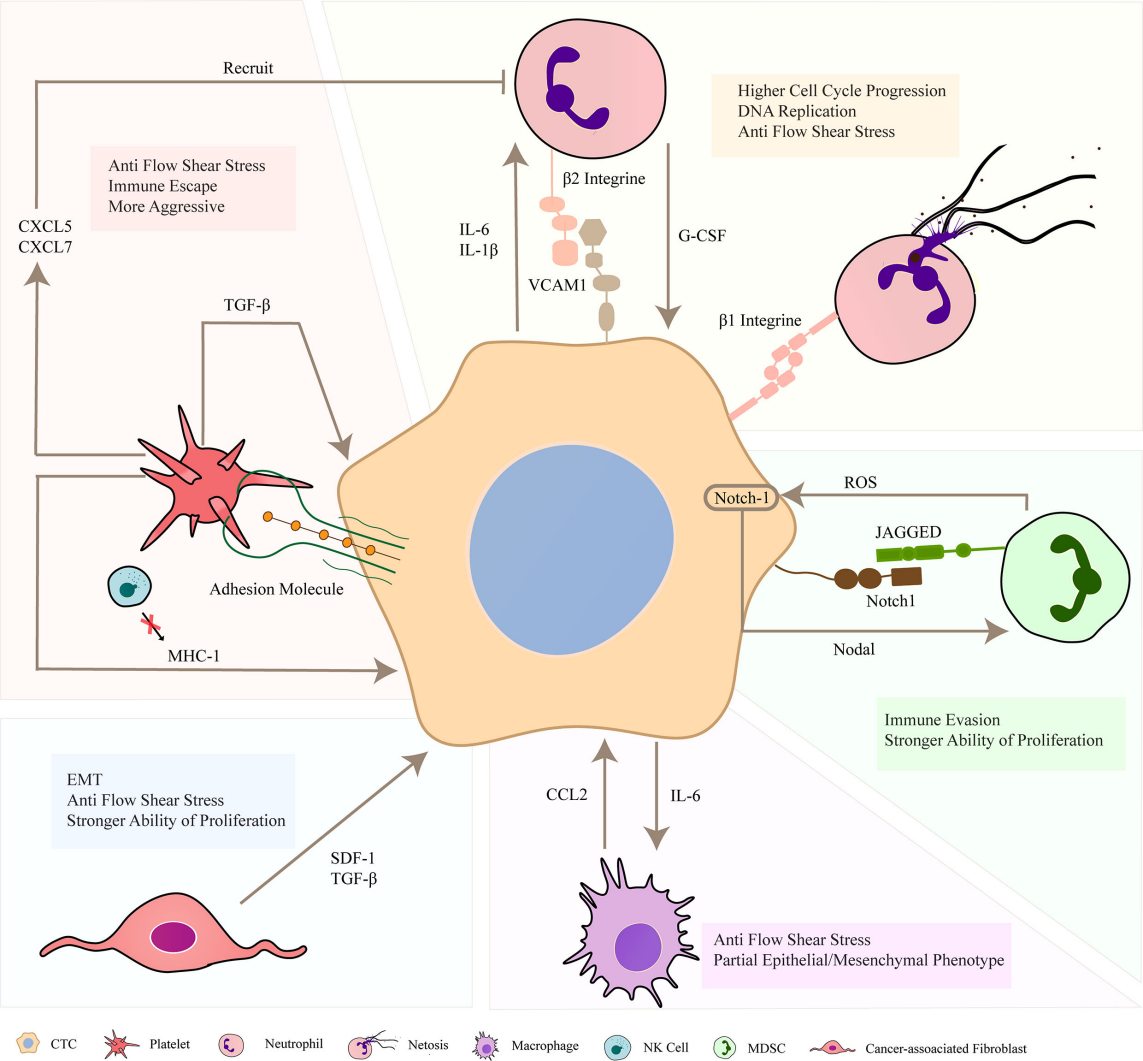
Formation of heterotypic circulating tumor cell (CTC) clusters. In the blood circulation, CTCs forms cell clusters with other non–tumor cells, such as neutrophils, platelets, macrophages, MDSCs and CAFs. These non–tumor cells can combine with cancer cells through key adhesion molecules to prevent apoptosis, and maintain the survival of CTCs *in vivo* through multiple ways. They can release some cytokines to strengthen the invasion and proliferation of cancer cells, and be more resistant to fluid shear stress (FSS). In addition, cancer cells also release related substances to act on non–tumor cells and recruit them to adhere to CTCs. In short, they jointly promote the occurrence and development of cancer in the blood system.

Throughout the development of metastatic tumors, CTCs are existing in the greatly complex vascular environment in which cancer cells interact with the outside world through various ways. In many types of cancer diseases, CTC clusters exhibit high heterotypic phenotype (69). Heterogeneity is a characteristic of malignant tumors. According to the related report, heterotypic phenotype can make cancer cells adapt to the changes of tumor microenvironment and is conducive to tumor progression (70). Some studies have shown that CTC clusters can be composed of some cancer cells and non–tumor cells. These non–tumor cells, such as platelets and neutrophils, will interact with CTCs in various ways to enhance the survival and metastasis of cancer cells. According to literature reports, the metastatic capability of heterotypic clusters is stronger than that of homotypic clusters (26). The interaction process between non-tumor cells and cancer cells is summarized in **Figure 3**.

### MDSCs

MDSCs are heterogeneous cells derived from bone marrow and are immature myeloid cells. MDSCs are usually accompanied by chronic inflammation, especially during advanced cancer (71). In the process of tumor progression, they can be recruited into the tumor microenvironment and exert immunosuppressive effects through various signaling pathways and mechanisms. On the one hand, MDSCs can produce ARG–1, iNOS, ROS to directly suppress host immunity (72). On the other hand, they can promote the proliferation of Treg cells and upregulate the immune checkpoint (PD–L1) to inhibit the immune response in an indirect way (73). After contacting with MDSCs, some cancer cells become flexible and leave their original growth “soil”, and then expand their “territory”, promoting cancer progression in distant organs (74).

In the meanwhile, MDSCs can exist and circulate in PB (75). In 2016, Yupei Zhao proposed the hypothesis that MDSCs adhered to CTCs would form a physical defense barrier to avoid immune surveillance and promote metastatic tumors (76). PMN–MDSCs have tumor–promoting effects at multiple levels and stages of metastasis, including inhibition of immune response, influence of tumor angiogenesis and characteristics of CSCs, and the formation of pre–metastasis niche. Some experiments have studied whether the interaction between CTC–MDSC clusters and MDSCs in the blood circulation is beneficial to tumor survival and metastasis through cluster analysis of CTC–MDSC clusters. Subsequently, scientists discovered melanoma patient CTC/PMN–MDSC heterotypic clusters captured by Parsortix microfluidic device. The ability of tumor propagation and metastasis was significantly enhanced in mice co–injected with CTCs and PMN–MDSCs. An important characteristic of PMN–MDSCs is the production of reactive oxygen species (ROS). ROS produced by PMN–MDSCs can upregulate the expression of Notch1 receptors on CTCs through the ROS–NRF2–ARE axis. In turn, Jagged1–expressing PMN–MDSCs help enhance Notch activation in CTCs through binding to the Notch1 receptor. In general, scientists discovered that the Nodal–Notch1–Jagged1 signaling pathway promoted changes in the characteristics and behaviors of CTCs (77). But the mechanism of how PMN–MDSCs and CTCs are connected, such as what kind of adhesion proteins contributes to the aggregation between them, remains to be further studied.

### Neutrophils

Neutrophils are the component of the innate immune system and the numerous white blood cells (WBCs) circulate in the human bloodstream. In patients with advanced tumor, neutrophils often accumulate in PB and metastatic tumor tissues, suggesting that neutrophils may be associated with metastatic tumors (78). Studies have found that neutrophils can promote the formation of pre–metastasis niche. Meanwhile, the increase in the number of neutrophils and the ratio of neutrophils to lymphocytes in PB can mark the poor prognosis of tumor patients (79). The interaction between β2 integrin of melanoma cells and ICAM1 of neutrophils was proven to mediate the combination of these two cells, which can deal with the damage of FSS (80).At the same time, melanoma cells were found to produce and secrete high levels of cytokine IL–8, which attracts neutrophils and increases the expression of β2 integrin in turn (81). In 2019, Nicola Aceto reported that in the blood samples from 70 breast cancer patients, CTC–WBC clusters were detected in nearly half of the cases, accounting for 3.4 percent of the total number of CTCs. Single–cell RNA sequencing revealed that most CTC–associated leukocytes were from the myeloid system, and further study showed that most of them were neutrophils. More importantly, patients with CTC–neutrophil clusters had poorer outcomes compared to those with single CTCs or homotypic CTC clusters in PB. CTC–neutrophil clusters show higher cell cycle progression and DNA replication programs than single CTCs, as confirmed by Ki67 expression. In addition, the experiment also verified that cancer cells expressing IL–6 and IL–1β could form a more proliferative CTC–neutrophils clusters than those lacking IL–6 and IL–1β receptors (82). Vascular cell adhesion molecular 1 (VCAM1), has been reported to be associated with cancer metastasis and EMT (83). In addition, VCAM1 was found to mediate the adhesion between CTCs and neutrophils (82). When treated with VCAM1 monoclonal antibody, the cellular junctions between CTCs and neutrophils would detach (84). Previous studies have found that melanoma cells that bind to neutrophils produced and secreted high levels of IL–8, attracting neutrophils and increasing β2 integrin expression rate. At the same time, ICAM1 on cancer cells and β2 integrin on neutrophils interact with each other to mediate their aggregation (85). In addition, cancer cells directly adhere to arrested neutrophils, which is mediated by Mac–1/ICAM1 (86). Recent studies have shown that CTCs analysis results and neutrophil–lymphocyte ratio can be combined to classify patients into different risk groups, which has certain clinical value (87).

NETs are extra–cellular networks released by neutrophils. They have been found to promote metastatic tumors as they have the abilities of enhancing the aggressiveness of cancer cells, regulating the pre–metastasis niche, promoting angiogenesis and enhancing vascular permeability by inducing EMT of cancer cells (88). CTCs can be wrapped in NETs to protect them from various attack of immune cells and FSS. β1 integrin expression on cancer cells and NETs is an important factor for the adhesion between them in PB (89). In addition to the fact that NETs can bind to CTCs in the bloodstream, studies have shown that NETs at remote metastatic sites could capture CTCs and promote tumor metastasis and colonization. A cancer cell surface receptor called CCDC25 was reported to bind specifically to NETs DNA (90). In summary, there are many interactions between neutrophils and CTCs that contribute to the development of tumor. The interaction between them needs to be further explored in the future.

### Platelets

Platelets are small clumps of cytoplasm released from mature megakaryocytes in bone marrow. Their main function is to clot and stop bleeding, and repair damaged blood vessels. The role of platelets in metastatic tumors was discovered as early as 1960s, and the thrombocytopenia can play a role in anti–tumor metastasis (75). In recent years, more and more studies have found that platelets are related to the disease progression of patients, and thrombocytosis is a common para–neoplastic phenomenon (91). Relevant clinical studies recently have revealed that platelets–covered CTC clusters can predict poor prognosis for pancreatic cancer (88).

When CTCs enter the circulatory system, they activate surrounding platelets. Then the platelets accumulate on the surface of CTCs and provide a physical barrier to protect cancer cells against external dangers. Platelets have been found to improve the tolerance of CTCs to FSS (92). Heterodimers composed of α subunits and β subunits are called integrins which play an important role in mediating cell connections, including their subtype α6β1, αvβ3, αIIbβ3 and α2β1, especially in adhesion between cancer cells and platelets (93, 94). One of the most important one is platelet membrane protein GPIIb/IIIa complex, which binds to adhesion proteins on the surface of cancer cells, thus mediating platelets–CTCs interaction (95). Toll–like receptors are expressed on the surface of platelets, which contribute to pathogen recognition and make platelets one of the main components of innate immunity (96). Surgical resection of solid tumor tissue will lead to more CTCs in PB. In a previous study, after external stimulation (surgical stress), platelet TLR4 led to phosphorylation of ERK5, and then promoted the aggregation between platelets and CTCs through surface integrin GPIIb/IIIa complex (97). Some types of CTCs can release von Willebrand factor (vWF) (98). Subsequently, vWF binds to the GPIbα subunit of platelet glycoprotein Ib–IX–V (GPIb–IX–V) to promote the aggregation and adhesion of platelets on the surface of CTCs (93). In addition, platelet integrin αIIbβ3 and the receptor of cancer cells, such as αvβ3, can mediate cancer cells–platelets aggregation. Despite helping overcome the shear stress resulting from blood flow, it can promote the CTCs adhesion on the vascular wall as well (99).

In addition to being able to adhere directly to CTCs, platelets also have immunosuppressive effects. NK cells are cytotoxic immune cells that kill cells expressing very little or even not expressing MHC–I molecules. In addition to physically protecting CTCs, platelets also transfer their MHC–I molecules to the surface of CTCs. When CTCs with MHC–I are recognized by NK cells, NK cells will not attack them, resulting in immune escape of CTCs (100).

Platelets can release a variety of growth factors, and TGF–β is one of them (101). TGF–β is thought as a role promoting metastasis by promoting the process of EMT and enhancing the aggressiveness of cancer cells (102). Direct effect of platelet–derived TGF–β has been shown as synergistically activating the TGFβ/SMAD and NF–κB pathways in cancer cells, leading to the transformation of cancer cells into an aggressive phenotype (103). Platelets in contact with CTCs can release chemokines of CXCL5 and CXCL7 to recruit neutrophils, which contributes to the formation of heterotypic clusters of CTCs and neutrophils (104).

### Macrophages

In tumor microenvironment (TME), macrophages, known as TAMs, can be polarized into macrophages with M1 phenotype, which can kill cancer cells by releasing a variety of cytotoxic molecules, while macrophages with M2 phenotype can contribute to tumor progression. In the early stage of tumor development, TAMs is conducive to stimulate immunity. But as the disease develops, it will become a tumor–promoting component (105).

Recent experiments in glioblastoma models have confirmed that the cell clusters formed by heterotypic combination of cancer cells and macrophages showed strong invasive ability (106). Studies have shown that macrophages could locate at the tumor site, then proliferate into the blood, and those macrophages are called circulating tumor–associated macrophage–like cells (CAMLs). In 10 percent of advanced patients, CAMLs were found to bind and attach to CTCs (107). There are many macrophage–CTC clusters in PB of patients with melanoma. Some researchers co–cultured cancer cells and macrophages, and found that cancer cells guided and accompanied by TAMs achieved partial epithelial/mesenchymal phenotype. In addition, CTCs that bind to macrophages can be more resistant to FSS, thus finally promoting metastatic tumors (108). In the experiment of colorectal cancer, TAMs–derived IL–6 activated the JAK2/STAT3 pathway and inhibited the tumor suppressor Mir–506–3p in cancer cells. Mir–506–3p was down–regulated in cancer cells, a key miRNA regulating FoxQ1, leading to increased FoxQ1 expression, which in turn resulted in the production of CCL2 and ultimately promoted macrophage recruitment. Inhibition of CCL2 or IL–6 terminated this chain of reactions. Subsequently, the migration ability of macrophages would be weakened, and the metastatic potential of CTCs would also be reduced (109). Clinically, macrophage–CTC clusters can be an independent prognostic marker for patients (110). Besides, there are direct cell–cell interactions between them. According to the report, the binding between CD47 on CTCs and inhibitory immune receptor signal regulatory protein alpha (SIRPα) on macrophages can induce the signal cascade of intracellular inhibition of phagocytosis (111).

### Cancer–associated fibroblasts

CAFs are defined as fibroblasts located in or near tumors. In numerous experiments, CAFs are considered to be fusiform cells that do not express marker genes of epithelium, endothelium and leukocyte (112). CAFs have the potential of regulating the interaction between cancer cells and other cells by secreting cytokines, growth factors, chemokines and exosomes, and can degrade matrix ECM by releasing matrix metalloproteinases (MMPs), while synthesizing new matrix proteins to promote tumor invasion and angiogenesis (113, 114). Before cancer cells enter the blood system, CAFs can promote the collective invasion of cancer cells that move within tracks in the extra–cellular matrix behind the CAFs (115).

In recent years, studies have found that CAFs exist in the blood circulation of tumor patients, and their number tested by liquid biopsy is correlated with tumor development and poor prognosis (116). CAFs, together with connected cancer cells, shed from the primary tumor lesion, then survive in the blood circulation, and proliferate in metastatic organs (117). CAFs in primary lesions can form CAF–CTC clusters with cancer cells. Studies have proved that CAFs can improve the ability of cancer cells to resist FSS by promoting cell adhesion and play an important role in maintaining the survival of CTCs in PB, thus facilitating the proliferation of aggregated cancer cells (118). Most metastatic cancer cells in the blood circulation and distant tissues are still closely connected with CAFs, and CD44 may be an important mediator of heteromorphic binding on the basis of scientific researches (119). In a mouse model of pulmonary adenocarcinoma, CAFs induce the formation of heterotypic clusters, thus promoting the formation of metastatic tumors. CAFs trigger EMT of CTC clusters through the release of SDF–1 and TGF–β, thus improving the ability of metastasis and invasion of CTC clusters (120). In a word, CAFs can protect CTC clusters through various mechanisms (121). More importantly, studies have shown that the level of CTC clusters and CAFs can be used as bio–markers to predict cancer recurrence (122). This shows that targeting CAF–CTC clusters has certain clinical significance.

## Potential therapeutic value of CTC clusters for tumor metastasis

Clinical patient diagnosis showed that patients with active cancer and acute ischemic stroke had more CTC clusters (123). Liquid biopsy is a test for biological fluids that may contain cancer derivatives, and cancer–derived substances may include CTCs and circulating tumor DNA (ctDNA). The most common sample is the fresh blood. Because the blood can be easily collected, liquid biopsy has become a powerful non–invasive tool for analyzing several cancer types. By combining morphology with CTCs counting, the liquid biopsy platform provides greater insights into the pathophysiology of disease (124). Besides, CTC–derived organoids could help capture tumor–related information to provide reference for clinically screening and testing promising drugs as well (125). Today, the detection of CTC clusters has been an independent indicator of relapse and mortality, and also associated with clinical–pathological surrogate markers of poor prognosis. In the meantime, as CTC clusters have become a recognized clinical biomarker for the severity and progression of tumor disease, the race is on to uncover the molecular mechanisms behind CTC clusters. Although metastasis is the leading cause of death in cancer patients and some drugs may shrink primary tumors to prevent metastasis, there are currently no drugs for clinical application that can directly treat or prevent metastasis by treating CTC clusters in PB (126). This is largely due to the lack of understanding of CTC clusters.

With the deepening of the researches, CTC clusters are considered to be the important factor affecting metastatic tumors, and the design and application of drugs targeting CTC clusters will help inhibit metastatic tumors effectively. In terms of the development and application of corresponding drugs, CTC clusters can be dissociated into single cells by drugs, which can inhibit metastatic tumors under physical factors such as FSS and immune cell strangulation, in view of the stronger survivability of CTC clusters compared to single CTCs. Many drugs have been found to have the potential to dissociate CTC clusters in related experiments. In a mouse model of breast metastatic tumors, scientists found that injecting urokinase–type plasminogen activator (uPA) into host animals effectively prevented clustering of CTCs and extended overall survival by about 20 percent compared with control animals (127). PEP06 is a novel cluster disaggregator, which destroys E–cadherin–mediated intercellular connections and thus significantly inhibits the migration, aggregation and colonization of fibronectin in oral squamous cell carcinoma in vitro (128). In addition, there are Na+/K+–ATPase inhibitors (compounds that inhibit the function of sodium–potassium pump), which can separate CTC clusters into individual cells and ultimately prevent metastatic tumors (52). HPSE inhibitors, which inhibit the progress of cancer by blocking the formation of CTC clusters, are currently being developed, and their functional mimics have been tested in clinical trials, but the further research is needed. Because HPSE inhibitors have the potential to prevent NK cells from infiltrating into the primary tumor, and increase the risk of metastasis. This leads to the limitations of the application of such drugs (129). Several drugs targeting EGFR have been developed and approved to treat tumor patients. A novel anti–EGFR monoclonal antibody (LA1) can effectively inhibit the formation of CTC clusters in vivo (33). But tumor is a multifactorial disease, so targeting a single target can lead to treatment failure. Most patients initially respond to treatment with EGFR inhibitors. However, patients usually develop drug resistance after 10–16 months of treatment (130). Therefore, the application of drugs that target two or more acting sites can be an effective strategy for treating tumors (131). According to the current research process, heterotypic CTC clusters are still in the initial stage. There are no clinical treatment methods and drugs for them despite their strong metastatic ability. However, I believe that in the future, with the deepening of researches, a series of drugs targeting heterotypic CTC clusters will be developed. In conclusion, dissociating homotypic CTC clusters into individual cancer cells can be a promising treatment for tumor metastasis. At the same time, the therapeutic methods targeting heterotypic CTC clusters, need to be discovered constantly based on the metastatic ability of heterotypic CTC clusters is stronger than that of homotypic CTC clusters.

## Discussion

Metastasis is the main cause of death in tumor patients. CTCs and CTC clusters are the “seeds” of metastasis and can be used as the research objects of liquid biopsy. Compared with single CTCs, and CTC clusters have stronger metastasis ability. Studies have shown that CTCs have an important relationship with the cells in PB. CTCs have closed interaction not only with platelets and neutrophils, as well as CAFs, macrophages and TAMs. These cells enable the CTCs to resist the damage of FSS in blood circulation and evade the detection of immune system, finally to leave the blood circulation and set up new sites of metastatic tumors. Although there have been a great deal of researches on these aspects in recent years, the current research phase is in its infancy and the deeper mechanisms and complex interactions have not been fully explained. In order to further study CTC clusters, it is necessary to optimize the enrichment and separation detection technologies, establish a unified standard, and improve the efficiency of machines. Secondly, we should further understand the oncological and biological characteristics of intercellular adhesion molecules that promote the formation of homotypic and heterotypic CTC clusters. Then, the mechanisms of formation and metastasis of CTC clusters should be explored to seek clinical therapeutic targets. Finally, the therapeutic targets of CTC clusters should be focused, and the occurrence and development of tumors before and after treatment are to be compared to find feasible treatment methods based on CTC clusters. Traditional Chinese medicine has been applied and booming for thousands of years. In recent decades, in order to determine the effectiveness of traditional Chinese medicine to treat cancer diseases, scientists have carried out extensive researches. Anti–tumor drugs relevant with invigorating the circulation of blood, can eliminate blood stasis and improve microcirculation. They can target hypercoagulable states and arteriovenous thrombosis in the blood of tumor patients, and also can treat complications such as thrombosis. Finally, they significantly improve the therapeutic effect of tumor and the quality of lives, so the application of traditional Chinese medicine, especially blood–activating and stasis–eliminating drugs, targeting CTC clusters to treat cancer diseases has a infinitely bright prospect. It is believed that in the near future, further researches will make people able to establish more new treatments and improve the survival rate of tumor patients.

## Author contributions

All authors listed have made a substantial, direct, and intellectual contribution to the work and approved it for publication.

## Funding

The work was funded by National Natural Science Foundation of China (No. 81673648, 81973734).

## Acknowledgments

This project was supported in part by National Natural Science Foundation of China, the Open Project of Chinese Materia Medica First–Class Discipline of Nanjing University of Chinese Medicine.

## Conflict of interest

The authors declare that the research was conducted in the absence of any commercial or financial relationships that could be construed as a potential conflict of interest.

## Publisher’s note

All claims expressed in this article are solely those of the authors and do not necessarily represent those of their affiliated organizations, or those of the publisher, the editors and the reviewers. Any product that may be evaluated in this article, or claim that may be made by its manufacturer, is not guaranteed or endorsed by the publisher.

## Abbreviations

CTCs, Circulating tumor cells; PB, Peripheral blood; EMT Epithelial–mesenchymal transition; EpCAM, Epithelial cell adhesion molecule; ECM, Extra–cellular matrix; FSS, Fluid shear stress; CSCs, Cancer stem cells; TM4SF5, Transmembrane 4 Six family members 5; FAK, Focal adhesion kinase; EGFR, Epidermal growth factor receptor; ICAM1, Inter–cellular adhesion molecule 1; TEM, Transendothelial migration; CDK6, Cyclin–dependent kinase 6 v; HPSE, Heparinase; Nox4, NADPH oxidase 4; K14, Keratin 14; PD–1, Programmed cell death 1; PD–L1, Programmed cell death ligand 1; HIF–1α, Hypoxia–inducible factor–1 alpha; DSG2, Desmoglein2; MDSCs, Myeloid–derived suppressor cells; TAMs, Tumor–associated macrophages; CAFs, Cancer–associated fibroblasts; ROS, Reactive oxygen species; WBCs White blood cells; VCAM1, Vascular cell adhesion molecular 1; vWF, Von Willebrand factor; GPIb–IX–V, Glycoprotein Ib–IX–V; PDGFs, Platelet–derived growth factors; TME, Tumor microenvironment; CAMLs, Circulating tumor–associated macrophage–like cells; SIRPα, Signal regulatory protein alpha; MMPs, Matrix metalloproteinases; ctDNA, Circulating tumor DNA; uPA, Urokinase–type plasminogen activator.
